# Altered responsiveness of BNST and amygdala neurons in trauma-induced anxiety

**DOI:** 10.1038/tp.2016.128

**Published:** 2016-07-19

**Authors:** O E Rodríguez-Sierra, S Goswami, H K Turesson, D Pare

**Affiliations:** 1 Center for Molecular and Behavioral Neuroscience, Rutgers State University, Newark, NJ, USA; 2 Department of Neuroscience, University of Texas Southwestern Medical Center, Dallas, TX, USA

## Abstract

A highly conserved network of brain structures regulates the expression of fear and anxiety in mammals. Many of these structures display abnormal activity levels in post-traumatic stress disorder (PTSD). However, some of them, like the bed nucleus of the stria terminalis (BNST) and amygdala, are comprised of several small sub-regions or nuclei that cannot be resolved with human neuroimaging techniques. Therefore, we used a well-characterized rat model of PTSD to compare neuronal properties in resilient vs PTSD-like rats using patch recordings obtained from different BNST and amygdala regions *in vitro*. In this model, a persistent state of extreme anxiety is induced in a subset of susceptible rats following predatory threat. Previous animal studies have revealed that the central amygdala (CeA) and BNST are differentially involved in the genesis of fear and anxiety-like states, respectively. Consistent with these earlier findings, we found that between resilient and PTSD-like rats were marked differences in the synaptic responsiveness of neurons in different sectors of BNST and CeA, but whose polarity was region specific. In light of prior data about the role of these regions, our results suggest that control of fear/anxiety expression is altered in PTSD-like rats such that the influence of CeA is minimized whereas that of BNST is enhanced. A model of the amygdalo-BNST interactions supporting the PTSD-like state is proposed.

## Introduction

Convergent findings from animal and human studies implicate a highly conserved network of brain structures in the expression of fear and anxiety.^1^ These include the medial prefrontal cortex, the bed nucleus of the stria terminalis (BNST), as well as the basolateral (BLA) and central (CeA) amygdala. Importantly, human functional imaging studies have revealed that many of these structures exhibit abnormal activity levels during symptom provocation in post-traumatic stress disorder (PTSD).^2, 3, 4^

However, BNST and the amygdala are in fact a collection of functionally heterogeneous nuclei that cannot be resolved with human functional imaging techniques. Thus, to shed light on the mechanisms that support the altered responsiveness of BNST and the amygdala in anxiety disorders, we used a well-characterized rat model of PTSD.^5^ In this model, Lewis rats are subjected to a species-relevant threatening experience, predatory threat, involving exposure to cat smell.^6, 7^ Following predatory threat, a subset (~50%) of susceptible rats (termed ‘PTSD-like’) develops severe and persistent (>2 weeks) behavioral manifestations of anxiety, including compromised exploratory behavior and increased startle.^6, 7^ Importantly, this model reproduces salient features of the human syndrome. For instance, PTSD is characterized by a fear extinction deficit that develops after trauma^8^ and a hippocampal-dependent allocentric spatial processing deficit that predates trauma.^9, 10^ The Lewis rat model of PTSD reproduces these two deficits, including their different temporal relationship to trauma.^5, 11, 12^

Therefore, using this model, we compared the intrinsic and synaptic responsiveness of BNST and amygdala neurons in resilient vs susceptible rats with patch recordings *in vitro*. This approach revealed that the PTSD-like state is associated with distributed but region-specific alterations in the synaptic responsiveness of BNST and amygdala neurons.

## Materials and methods

### Subjects and experimental timeline

**Figure 1:**
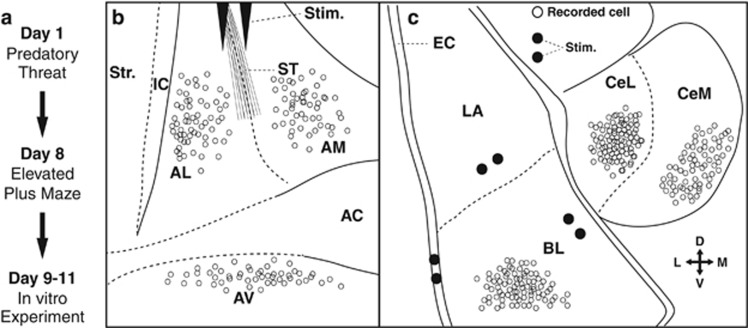
Experimental paradigm and recording sites. (**a**) Timeline of the experiments. (**b**) Scheme showing stimulation (stim.) and recording (circles) sites for the bed nucleus of the stria terminalis (BNST) experiments. A pair of stimulating tungsten electrodes was positioned in the stria terminalis (ST). Visually-guided patch-clamp recordings were performed in three different BNST-A regions: anterolateral (AL), anteromedial (AM) and anteroventral (AV). (**c**) Scheme showing stimulation (filled circles) and recording (empty circles) sites for the amygdala experiments. Patch clamp recordings were performed in the basolateral (BL) nucleus, as well as the lateral (CeL) and medial (CeM) sectors of CeA. Stimulating electrodes were positioned in the external capsule (EC) and lateral amygdala (LA) when recording BL neurons or in the BL nucleus when studying CeA neurons. For clarity, CeL neurons recorded in the glutamate uncaging experiments were not included. However, they were recorded in the same part of CeL as the cells currently depicted. Cross on lower right indicates orientation of the schemes (D, dorsal; L, lateral; M, medial; V, ventral). AC, anterior commissure; IC, internal capsule; Str., striatum.

Procedures were approved by the Institutional Animal Care and Use Committee of Rutgers State University and complied with the Guide for the Care and Use of Laboratory Animals (Department of Health and Human Services). Adult male Lewis rats (200–225 g) were first subjected to predator threat and, 7 days later, tested on the elevated plus maze (EPM), a well-accepted behavioral test of anxiety (Figure 1a). Next, based on their behavior in the EPM, rats were sorted into ‘PTSD-like’ or ‘resilient’ phenotypes as described below. Then, 1–3 days later rats were anesthetized, their brains extracted and coronal slices prepared for visually-guided patch-clamp neuronal recordings in BNST or the amygdala (Figures 1b and c). Investigators were blind to the rats’ phenotype.

### Predatory threat

The threatening experience consisted of a single exposure (10 min) to soiled cat litter in a plastic rat cage with a mesh top. The litter was obtained from male cats (sifted for stools; 2-day use period) and included the odor of cat feces, urine, hair and skin. Predator odors are such potent stressors that they can be used as unconditioned stimuli to drive Pavlovian fear learning.^13, 14^

### EPM and criteria used to classify rats as resilient vs PTSD-like

Rats were placed at the EPM’s center (Supplementary Methods), facing an open arm and allowed to explore the apparatus for 5 min. Behavior was recorded and scored offline. All four paws had to be into the arm to be classified as an arm entry. Consistent with prior work,^6, 7, 11, 12^ rats with extremely compromised exploratory behavior, that is spending all of the available time in the EPM’s closed arms, were classified as PTSD-like. Rats that explored the open arms for any amount of time were classified as resilient. Predatory threat exposure increases the incidence of the PTSD-like phenotype in the EPM: from ~10% in Lewis rats exposed to clean litter to ~50% after predatory threat.^6, 7, 11^ However, predatory threat does not increase anxiety in all rats but causes the emergence of an extremely anxious phenotype *in a subset of susceptible Lewis rats.*^11^ Indeed, comparisons of time spent in the open arms in rats exposed to soiled vs clean litter, but excluding subjects that did not explore the open arms, revealed no group differences.^11^ Moreover, resilient rats displayed a higher incidence of other behaviors suggestive of resilience.^11^

### *In vitro* slice preparation

Rats were anesthetized with Avertin (300 mg kg^−1^, i.p.) and perfused transcardially with a cold modified artificial cerebrospinal fluid (aCSF) detailed in the Supplementary Methods. Brains were cut in 300-μm-thick coronal slices using a vibrating microtome while in the above solution. Slices were then transferred to an incubating chamber for at least 1 h at room temperature in a control aCSF (Supplementary Methods). The slices were transferred one at a time to a recording chamber perfused with the latter solution (10 ml min^−1^). Before beginning the recordings, we gradually increased the chamber temperature to 32 °C.

### Electrophysiology

We obtained visually-guided whole-cell recordings using pipettes filled with a standard intracellular solution (Supplementary Methods). Membrane potential (*V*_m_) values were corrected for the liquid junction potential (10 mV with this solution). We only considered cells that had stable resting potentials negative to −60 mV and generated overshooting action potentials. Electroresponsive properties were characterized by studying the cells’ responses to graded series of current pulses (±20-pA steps, 500 ms) applied from rest, –65 or –80 mV. The linear portion of current–voltage plots was used to estimate the cells’ input resistance. To activate synaptic inputs to BNST cells, stimulating electrodes were positioned in the stria terminalis (ST; Figure 1b). To activate synaptic inputs to amygdala neurons, stimulating electrodes were positioned at one of three sites: in the external capsule and lateral amygdala (LA) when recording neurons in the basolateral (BL) nucleus or in BL when studying CeA neurons. Unless otherwise noted, electrical stimuli (100 μs) were delivered at a low frequency (0.1 Hz), in a range of intensities, and from a *V*_m_ of –65 mV. At least three stimuli were delivered at each intensity and responses averaged.

Presumably because the stimulation and recording sites were closer in the BNST than amygdala experiments, high-intensity ST stimuli elicited direct spikes in many of the recorded BNST cells. Thus, we used a lower range of stimulation intensities in the BNST (100–600 μA) than the amygdala (100–800 μA) experiments. When electrical stimuli elicited direct spikes, data obtained at that intensity and above was not considered for that cell. See Supplementary Methods for the criteria used to distinguish, direct, orthodromic and antidromic action potentials. Finally, when the stimuli elicited a mixture of sub- and supra-threshold responses, more stimuli were applied.

### Glutamate uncaging

Postsynaptic responsiveness to glutamate was assessed using glutamate uncaging. In these experiments, glutamate (4-Methoxy-7-nitroindolinyl-caged-l-glutamate, 1.0 mm; Tocris Bioscience, Bristol, UK) was added to the aCSF. Glutamate was uncaged by applying ultraviolet light pulses (5–30 ms, steps of 5 ms) over the recorded cell. Responsiveness to uncaged glutamate was assessed from a membrane potential of –80 mV, as determined by intracellular current injection. The ultraviolet light stimuli were delivered by a LED source (365 nm, 60 mW; 0.1 Hz; CoolLED, Andover, UK) via a × 60 immersion objective.

### Data analyses and statistics

The data were analyzed offline with the software IGOR (Wavemetrics, Portland, OR, USA), clampfit (Axon instruments, Foster City, CA, USA), and custom software written using Numpy and Scipy (http://www.scipy.org). Values are expressed as means±s.e.m. All statistical tests are two-sided. In all cases, all available cells, trials and subjects are included in the statistical analyses, as appropriate. The tests aimed to determine whether there were differences between neurons recorded in the two rat phenotypes. Depending on sample sizes, we used *X*^2^- or Fisher's exact tests to determine if phenotypic differences in the incidence of different cells types were significant. To compare their electroresponsive properties, we used non-parametric Kruskal–Wallis one-way analysis of variance (ANOVA). Since the synaptic responsiveness data were distributed normally, we used repeated measures ANOVAs for these comparisons.

## Results

We subjected 166 Lewis rats to predatory threat and tested them on the EPM 1 week later. Subjects with extremely compromised exploratory behavior (no time in the open arms of the EPM) were categorized as PTSD-like (48%) and the others as resilient (52%). One to three days later, we performed visually-guided patch-clamp recordings of neurons in different regions of BNST or the amygdala (Figures 1b and c).

### BNST experiments

We focused on the anterior BNST region (BNST-A) because it has been most frequently implicated in anxiety.^15^ However, BNST is a collection of ~15–20 nuclei with much disagreement regarding their exact number and location.^16, 17^ Compounding this problem, individual BNST nuclei cannot be identified with precision in living slices. Therefore, we used a simpler parcellation of BNST-A in three regions, based on the position of major fiber bundles (Figure 1b): the anterior commissure dividing the BNST-A in dorsal and ventral (BNST-AV) sectors, and the intra-BNST component of the ST subdividing the dorsal portion in medial (BNST-AM) and lateral (BNST-AL) regions. The following is based on samples of 61 BNST-AL cells (resilient, *n=*33; PTSD-like, *n=*28) recorded in 38 rats, 52 BNST-AM cells (resilient, *n=*27; PTSD-like, *n=*25) recorded in 32 rats, and 50 BNST-AV neurons (resilient, *n=*24; PTSD-like, *n=*26) recorded in 30 rats.

### Incidence, passive properties and spike characteristics of BNST-A neurons in resilient vs PTSD-like rats

BNST-A contains at least five different physiological cell types.^18, 19, 20, 21^ In decreasing order of incidence, they are low-threshold bursting (LTB) neurons, regular spiking (RS) cells, neurons expressing a fast inward rectifying K^+^ conductance (fIR), spontaneously active cells and late-firing neurons. Unfortunately, with the exception of the rare fIR neurons expressing CRF,^22^ there is no consistent relationship between the physiological properties of BNST-A neurons and their transmitter content (reviewed in ref. 23). Most BNST-A neurons are GABAergic neurons.^24, 25, 26, 27^ A few glutamatergic cells have also been identified in BNST-AM and AV,^27, 28^ where they are intermingled with the prevalent GABAergic neurons. Thus, it is likely that the vast majority of the cells we recorded belong to the prevalent class of GABAergic neurons.^29^

We found no phenotype-related variations in the incidence of the physiological cell types (*X*^2^-tests, *P*’s⩾0.51; Supplementary Table 1), which appeared to fall in the normal range previously reported in naive rats.^18, 19, 20, 21^ Moreover, we detected no phenotype-related differences in these cells’ passive properties and spike characteristics (Kruskal–Wallis one-way ANOVAs, *P*’s ⩾0.05; Supplementary Tables 2–7). Because three of the five cell types are rare, subsequent comparisons focused on the prevalent RS and LTB cells.

### Synaptic responsiveness of BNST-A neurons in resilient vs PTSD-like rats

To study the synaptic responsiveness of BNST-A neurons, we positioned stimulating electrodes in the ST (Figure 1b). This fiber bundle carries inputs from the main afferent of BNST, the amygdala. Indeed, the BNST-A receives very strong glutamatergic and GABAergic inputs from the BLA and CeA, respectively (Supplementary Figure 1).^25, 30, 31^ Therefore, from a *V*_m_ of −65 mV, we activated these axons at a low frequency (0.1 Hz) by delivering brief (100 μs) electrical stimuli (0.1–0.6 mA) through the ST electrodes. As described below, we found marked region-specific differences in the synaptic responsiveness of BNST-A neurons between the two rat phenotypes. However, within each region, the various physiological cell types exhibited the same trends. Thus, for simplicity, the results obtained in the different classes of neurons are pooled below.

### BNST-AL neurons

**Figure 2:**
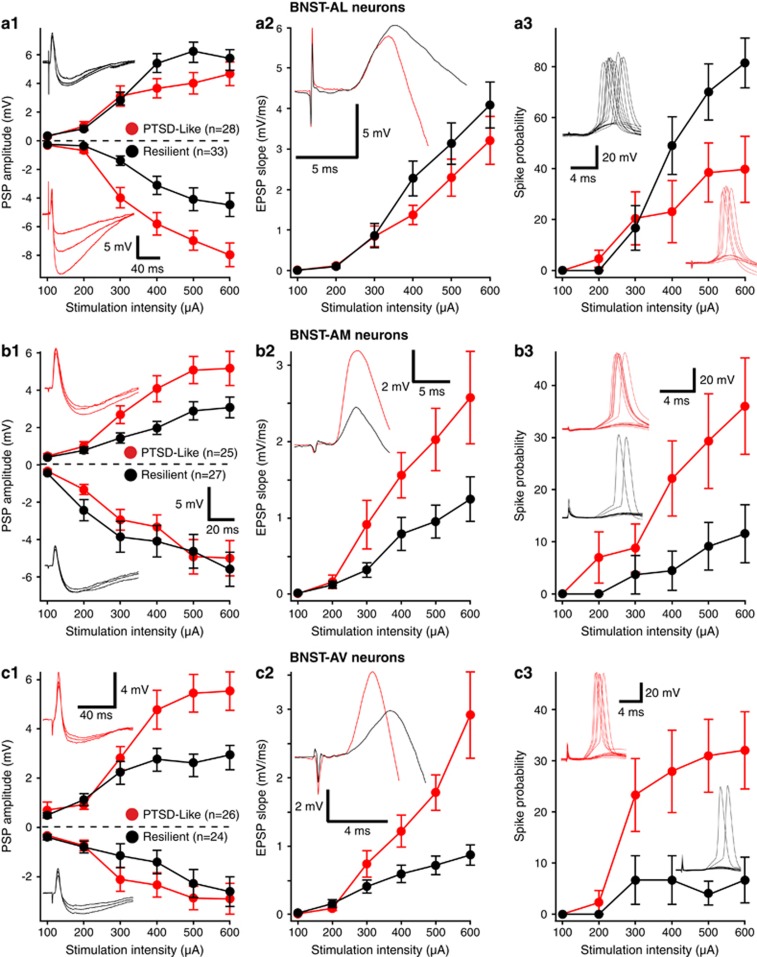
Synaptic responsiveness of BNST neurons to ST stimuli in resilient (black) and PTSD-like (red) rats. Neurons recorded in BNST-AL (**a**), BNST-AM (**b**) or BNST-AV (**c**). The *x*-axis represents stimulation intensity, whereas the *y*-axis shows (1) the amplitude of evoked EPSPs and IPSPs (positive and negative values, respectively), (2) EPSP slopes (measured in the first 2 ms) and (3) the proportion of trials eliciting orthodromic spikes. Insets show representative examples of evoked responses for neurons recorded in resilient (black) and PTSD-like rats (red). Error bars indicate s.e.m. AL, anterolateral; AM, anteromedial; AV, anteroventral; BNST, bed nucleus of the stria terminalis; EPSP, excitatory postsynaptic potential; IPSP, inhibitory postsynaptic potentials; PTSD, post-traumatic stress disorder; ST, stria terminalis.

The synaptic responsiveness of BNST-AL neurons was lower in PTSD-like than resilient rats (Figure 2a). This difference was mostly due to the higher amplitude of ST-evoked inhibitory postsynaptic potentials (IPSPs) in cells from PTSD-like rats (Figure 2a1; ANOVA, F_(1,59)_=8.821, *P=*0.006). Although there was a trend for ST-evoked excitatory postsynaptic potentials (EPSPs) to have lower amplitudes in neurons from PTSD-like than resilient rats (Figure 2a1), it did not reach significance (F_(1,59)_=2.4, *P=*0.07). Consistent with this, the slope of ST-evoked EPSPs did not differ significantly between the two rat phenotypes (Figure 2a2). Despite the similar properties of ST-evoked EPSPs in the two phenotypes, the likelihood that ST stimuli would elicit orthodromic spiking was significantly higher in neurons from resilient than PTSD-like rats (Figure 2a3; F_(1,59)_=5.803, *P=*0.019). Overall, these results suggest that differences in the potency of ST-evoked inhibition contribute to reduce the orthodromic responsiveness of BNST-AL neurons in PTSD-like relative to resilient rats.

### BNST-AM neurons

Opposite to BNST-AL neurons, the responsiveness of BNST-AM cells was higher in PTSD-like than resilient rats. Indeed, the amplitude (Figure 2b1) and slope (Figure 2b2) of ST-evoked EPSPs were significantly higher in neurons recorded from PTSD-like than resilient rats (EPSPs, F_(1,50)_=5.762, *P=*0.02; Slope, F_(1,50)_=4.95, *P=*0.03), with no difference in IPSP amplitudes (Figure 2b1; F_(1,50)_=0.179, *P=*0.67). Accordingly, the probability that ST stimuli would elicit supra-threshold responses was significantly higher in PTSD-like than resilient rats (Figure 2b3; F_(1,50)_=5.09, *P=*0.028).

### BNST-AV neurons

Similar to BNST-AM cells, but opposite to BNST-AL neurons, the responsiveness of BNST-AV cells was *higher* in PTSD-like than in resilient rats. This was evidenced by the significantly higher amplitude (Figure 2c1) and slope (Figure 2c2) of ST-evoked EPSPs in PTSD-like rats (EPSPs, F_(1,48)_=9.65, *P=*0.003; Slope, F_(1,48)_=9.309, *P=*0.004), with again no difference in IPSP amplitudes between the two rat phenotypes (Figure 2c1; F_(1,48)_=0.425, *P=*0.51). Paralleling these results, spiking probability in response to ST stimuli was significantly higher in PTSD-like than resilient rats (Figure 2c3; F_(1,48)_=11.51, *P=*0.001).

### Mechanisms underlying differences in the synaptic responsiveness of BNST-AM and AV cells

To determine whether the phenotypic differences in EPSP properties described above are dependent on a presynaptic mechanism, we compared the amount of paired-pulse facilitation (PPF) in the two groups of BNST-AM and AV neurons (Supplementary Figure 2). In this analysis,^32^ two identical stimuli are applied in rapid succession. PPF magnitude is inversely proportional to transmitter release probability: manipulations that increase release probability decrease PPF and conversely.^33, 34, 35^ Therefore, in the presence of picrotoxin (100 μm) and in voltage-clamp mode, we applied two ST stimuli in rapid succession and computed the ratio of the EPSC amplitude they elicited (EPSC2/EPSC1) in BNST-AM (Supplementary Figure 2A), and AV (Supplementary Figure 2B) neurons in the two phenotypes. In both BNST regions, the paired-pulse ratio did not differ significantly between groups (Supplementary Figure 2; *t*-tests, AM, *P=*0.8; AV, *P=*0.1).

### Amygdala experiments

We recorded 82 BL neurons (resilient, *n=*38; PTSD-like, *n=*44) from 32 rats; 138 central lateral (CeL) neurons (resilient, *n=*69; PTSD-like, *n=*69) from 37 rats; and 71 central medial (CeM) neurons (resilient, *n=*26; PTSD-like, *n=*45; Figure 1c) from 22 rats. CeL and CeM are considered separately because they form contrasting connections with fear output networks. While CeM contributes extensive projections to various brainstem fear effector structures, CeL outputs are mostly limited to the parabrachial nucleus.^36, 37, 38^ However, CeL regulates fear expression via its GABAergic projections to CeM^39, 40, 41, 42^ and BNST.^31^

### Passive properties and incidence of different cell types in resilient vs PTSD-like rats

#### BL neurons

**Figure 3:**
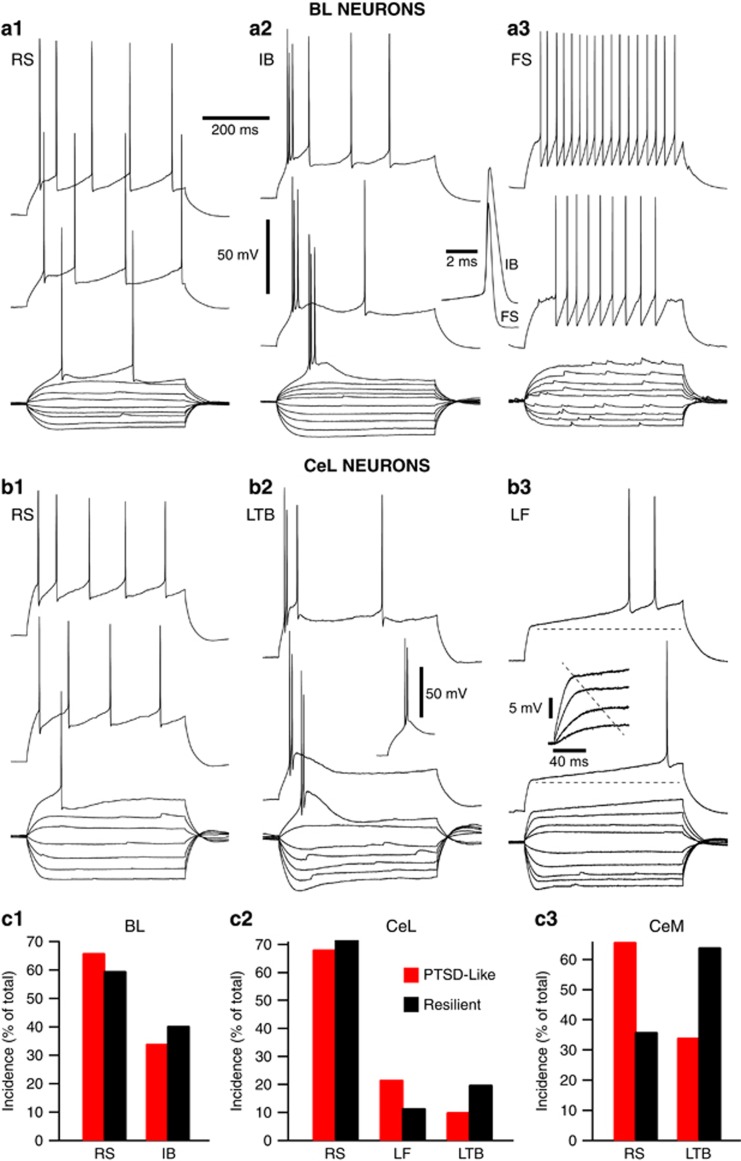
Incidence of different physiological classes of amygdala neurons. Voltage responses of six different cells to negative and positive current pulses of progressively increasing amplitude (current step increments of −0.04 nA for negative and sub-threshold positive pulses; current step increments of 0.02 nA for supra-threshold pulses). Unless otherwise noted, stimuli were applied from a membrane potential of −80 mV, as determined by steady intracellular current injection. (**a**) In BL, three types of neurons could be distinguished: regular spiking (RS; **a1**), intrinsically bursting (IB; **a2**) and fast spiking (FS; **a3**). Inset between **a2** and **a3** overlays action potentials generated by FS and IB cells. (**b**) In CeL, three types of neurons could be distinguished: RS (**b1**), low-threshold bursting (LTB; **b2**) and late firing (LF, **b3**). Inset below top trace of **b2** shows rebound spike doublet generated at the break of a −0.2 nA hyperpolarizing pulse applied from −65 mV. Inset in **b3** shows change in the time course of voltage responses to sub-threshold depolarizing current pulses. Voltage and time calibrations between A1 and A2 apply to all panels with the exception of insets. (**c**) Phenotypic variations in the incidence of physiological cell types. (**c1–3**) Amygdala neurons recorded in BL (**c1**), CeL (**c2**) and CeM (**c3**). Sample sizes: (**c1**) BL neurons from resilient (*n=* 38) and PTSD-like rats (*n=*44); (**c2**) CeL neurons from resilient (*n=*69) and PTSD-like rats (*n=*69). (**c3**) CeM neurons from resilient (*n=*25) and PTSD-like rats (*n=*41). BL, basolateral; CeL, central lateral; CeM, central medial; PTSD, post-traumatic stress disorder; RS, regular spiking.

Consistent with prior work,^43, 44, 45, 46^ we classified BL neurons as putative projection cells (Figure 3a1) or interneurons (Figure 3a3) based on their contrasting electroresponsive properties (reviewed in refs 47, 48). BL neurons were classified as projection cells when they displayed spike frequency adaptation during depolarizing current pulses and generated action potentials of comparatively long duration (⩾0.8 ms at half amplitude). Given the heterogeneous firing patterns of BL interneurons,^49, 50, 51^ we relied primarily on spike duration to identify these cells (⩽0.6 ms at half amplitude). Because a very low proportion of recorded cells met this criterion, they are not considered further. Consistent with previous findings,^45^ two types of BL projection cells were distinguished based on their responses to depolarizing current pulses: cells generating only single spikes (Figure 3a1), hereafter termed RS cells, and neurons generating spike doublets or bursts (Figure 3a2), hereafter termed intrinsically bursting. The incidence of RS and intrinsically bursting neurons did not vary between resilient and PTSD-like rats (Fisher’s exact test, *P=*0.8230; Figure 3c1). Whether we considered the two types of projection cells together or separately, spike duration, amplitude and threshold did not vary significantly as a function of the rats’ phenotypes, nor did their resting potential, time constant or input resistance (*t*-tests, *P*’s>0.1, Supplementary Table 8).

#### CeL neurons

Consistent with prior studies in rats,^39, 52, 53^ we identified three main cell types in CeL, based on variations in the temporal dynamics of current-evoked spiking: RS (Figure 3b1), LTB (Figure 3b2) and late-firing (LF, Figure 3b3). However, their incidence did not vary significantly between resilient vs PTSD-like rats (Figure 3c2; *X*^2^-test, *χ*^2^=3.11, *P=*0.211). Moreover, whether we considered the three cell types together or separately, the two behavioral phenotypes were not associated with differences in the spiking or passive properties of CeL neurons (*t*-tests, *P*’s>0.08; Supplementary Table 9).

#### CeM neurons

In prior studies,^52, 54^ the same physiological classes of neurons identified in CeL were found in CeM, albeit with differences in their relative incidence. Our results in CeM matched these earlier findings with the exception that we encountered no LF cells. In contrast with BL and CeL neurons, marked differences in the incidence of CeM cell types were observed as a function of the rats’ phenotype (Figure 3c3). In particular, RS cells prevailed in PTSD-like rats whereas the incidence of LTB neurons was higher in resilient rats (Fisher's test, *P=*0.017). However, comparing the spike or passive properties of CeM neurons (Supplementary Tables 10–12) revealed no significant differences between PTSD-like and resilient rats (*P*’s⩾0.09).

### Synaptic responsiveness of amygdala neurons in resilient vs PTSD-like rats

#### BL neurons

**Figure 4:**
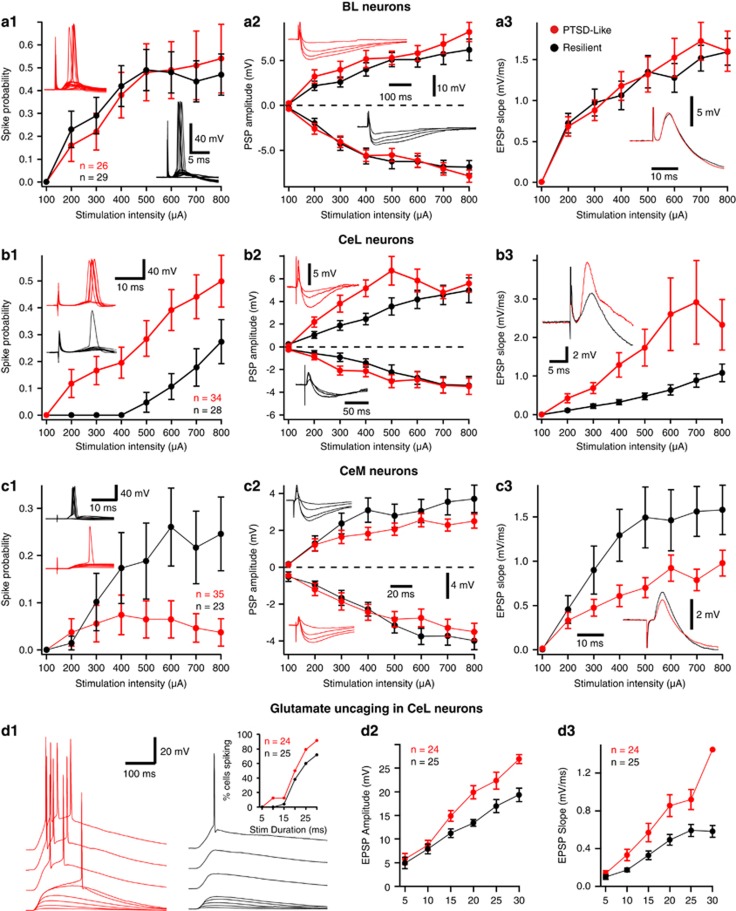
Synaptic responsiveness of BL (**a**), CeL (**b**) and CeM (**c**) neurons in resilient (black) and PTSD-like (red) rats. In all panels, the *x*-axis represents stimulation intensity whereas the *y*-axis shows (1) proportion of trials eliciting orthodromic spikes, (2) the amplitude of evoked EPSP and IPSP (positive and negative values, respectively), as well as (3) EPSP slopes (measured in the first 2 ms). Error bars indicate s.e.m. Stimulation sites were LA (**a1**), EC (**a2**, **a3**), BL (**b**,**c**). Insets show representative examples of evoked responses for neurons recorded in resilient (black) and PTSD-like rats (red). (**d**) Responses of CeL neurons to uncaged glutamate vary as a function of the rats’ phenotype. (**d1**) Representative examples of responses elicited by UV light pulses of gradually increasing duration (from 5 ms at bottom to 45 ms at top). Inset: proportion of cells spiking (*y*-axis) as a function of UV stimulus duration (*x*-axis). Beyond 30 ms, all cells fired. (**d2**) Peak amplitude of EPSPs elicited by glutamate uncaging (*y*-axis) as a function of UV stimulus duration (*x*-axis). In this and the next panel, all supra-threshold responses were excluded, resulting in progressively diminishing *n*’s with UV stimuli of increasing durations (see inset of **b1**). (**d3**) Slope of EPSPs elicited by glutamate uncaging (*y*-axis) as a function of UV stimulus duration (*x*-axis). Error bars indicate s.e.m. BL, basolateral; CeL, central lateral; CeM, central medial; EC, external capsule; EPSP, excitatory postsynaptic potential; IPSP, inhibitory postsynaptic potentials; LA, lateral amygdala; PTSD, post-traumatic stress disorder; UV, ultraviolet.

BL receives excitatory inputs from several cortical regions^55^ and the LA.^56, 57^ To test whether the responsiveness of BL neurons to these inputs differed between the two phenotypes, we positioned stimulating electrodes in the external capsule, which carries most cortical axons ending in BL, and the ventral part of LA. We then compared the responses elicited by electrical stimuli (100 μs; 0.1–0.8 mA) delivered at these two sites (resilient, *n=*34; PTSD-like, *n=*24). Irrespective of the stimulation site, no phenotypic differences were seen in the proportion of stimuli eliciting spikes (Figure 4a1; F_(1,56)_=0.526, *P=*0.471), in the amplitude of evoked EPSPs or IPSPs (Figure 4a2; EPSPs, F_(1,56)_=0.743, *P=*0.512; IPSPs, F_(1,56)_=0.239, *P=*0.689) or in the slope of EPSPs (Figure 4a3; F_(1,56)_=0.6872, *P=*0.356).

#### CeL neurons

BL neurons contribute glutamatergic inputs to CeL and CeM.^57, 58, 59, 60^ Therefore, we compared the properties of BL-evoked synaptic responses in the two phenotypes. CeL cells (resilient, *n=*28; PTSD-like, *n=*34) displayed marked differences in synaptic responsiveness as a function of phenotype (Figure 4b). First, the proportion of BL stimuli eliciting spikes was significantly higher in PTSD-like than resilient rats (ANOVA, F_(1,60)_=8.693, *P=*0.0045; Figure 4b1) and this effect was seen in both RS and LF neurons. Consistent with the higher probability of orthodromic spiking in PTSD-like rats, CeL EPSP (but not IPSP) amplitudes (Figure 4b2; ANOVA, F_(1,60)_=4.75, *P=*0.033) and slopes (Figure 4b3; ANOVA, F_(1,60)_=4.192, *P=*0.045) were higher in PTSD-like than resilient rats, particularly in an intermediate range of stimulation intensities (0.2–0.5 mA).

To determine whether the increased synaptic responsiveness of CeL neurons in PTSD-like rats was due to pre- or postsynaptic factors, we compared properties of PPF in the two groups (Supplementary Figure 3), as in the BNST experiments. However, the paired-pulse ratio did not differ significantly (*t*-test, *t*=−0.302, df=32, *P=*0.765; PTSD=1.55±0.064, resilient=1.52±0.068).

Therefore, to test whether a difference in the postsynaptic sensitivity of CeL neurons to glutamate mediates the differences in BL-evoked responses, we used photic uncaging of glutamate. In this approach, slices are bathed in an aCSF solution containing caged glutamate (1.0 mm). Ultraviolet light stimuli (5–30 ms), centered over the soma of the recorded cell, are applied to uncage glutamate. Figure 4d1 illustrates representative examples of responses to stimuli of progressively increasing duration (bottom to top) in CeL neurons from PTSD-like (red, left) and resilient (right, black) rats. As in these representative examples, the average amplitude (Figure 4d2) and slope (Figure 4d3) of EPSPs elicited by uncaged glutamate was significantly higher in CeL cells from PTSD-like than resilient rats (PTSD-like, *n=*24; resilient, *n=*25; amplitude, F_(1,47)_=30.28, *P*<0.001; slope, F_(1,47)_=13.12, *P=*0.004). Note that these differences were detected despite the fact that the analyses excluded trials where cells fired in response to uncaged glutamate. At all stimulation intensities, a higher proportion of supra-threshold trials were seen in CeL neurons from PTSD-like rats (Figure 4d1, inset).

#### CeM neurons

Opposite to the results obtained in CeL, CeM neurons from PTSD-like rats had a lower synaptic excitability. First, the proportion of BL stimuli eliciting spikes (Figure 4c1) were significantly lower in CeM cells from PTSD-like than resilient rats (ANOVA, F_(1,57)_=4.5, *P=*0.033). Similarly, EPSP slopes (Figure 4c3) were significantly lower in the PTSD-like group (F_(1,57)_=6.028, *P=*0.017). EPSP amplitudes (Figure 4c2) displayed a parallel trend but group differences did not reach significance (F_(1,57)_=2.947, *P=*0.09).

## Discussion

Using patch recordings in brain slices kept *in vitro*, we studied the intrinsic and synaptic responsiveness of BNST and amygdala neurons in a rat model of PTSD. By comparing BNST and amygdala neurons in resilient and susceptible rats, we observed region-specific differences in their synaptic excitability. Below, we discuss the significance of these observations in light of prior work on the role and connections of the amygdala and BNST.

### Limitations of the *ex vivo* approach and validity of the model

Although physiological studies in brain slices have substantial analytical power, they also have some limitations. On the negative side, many connections, particularly those involving distant structures, are lost. Consequently, network phenomena that might play an important role in PTSD cannot be studied with this approach, raising the possibility that some of the differences we observed *in vitro* are mitigated or enhanced by activity in BNST or amygdala afferents.

On the positive side, the *ex vivo* approach allows one to study nuclei-specific alterations in physiological properties; BNST and the amygdala do not have to be treated as undifferentiated structures because of insufficient spatial resolution. Also, the *ex vivo* paradigm allows identification of phenotypic differences in neuronal excitability, independently of emotion and cognition. This contrasts with human imaging studies where neuronal activity and emotions are inextricable. However, it is impossible to determine whether the differences we observed predated exposure to predator threat or emerged as a result of this experience. Related to this, it is conceivable that the susceptibility of PTSD-like rats to predator threat is due to their increased responsiveness to olfactory stimuli relative to resilient rats.

### Altered BNST and amygdala responsiveness in PTSD-like vs resilient rats

Early lesion and inactivation studies that lacked the spatial resolution to selectively affect different BNST sub-regions led to the view that BNST activity promotes the development of long-lasting anxiety states.^61, 62, 63, 64, 65^ However, more recent work indicates that BNST is functionally heterogeneous.^66, 67^ First, much data indicates that CRF neurons of the oval nucleus, located in the dorsal half of BNST-AL, exert an anxiogenic influence. For instance, stressors increase CRF mRNA expression in BNST-AL (reviewed in ref. 23) and infusing CRF in BNST is anxiogenic.^68^ Moreover, anxiety is reduced after chemogenetic inactivation of CRF cells^69^ or optogenetically silencing BNST-AL cells expressing D1-receptors,^66^ thought to be only expressed by CRF cells.^23^ However, it is currently unclear how CRF cells promote anxiety. Since they do not project to the pituitary, their influence probably involves a modulation of synaptic transmission in BNST itself^23^ or in their targets.

Related to this, CRF cells account for a minority of BNST-AL neurons^22^ raising the question of what is the role of the prevalent non-CRF GABAergic neurons? It should be noted that most of our BNST-AL cells presumably belonged to this group since only ~10% of our sample were fIR neurons. Mounting evidence suggests that these non-CRF cells are inhibited during fear and anxiety. Indeed, a peptide that when infused in BNST, potentiates acoustic startle and increases neuronal activity in BNST-AL’s brainstem targets^70^ actually inhibits non-CRF BNST-AL neurons *in vitro.*^71^ Moreover, BNST-AL neurons acquire inhibitory responses to conditioned stimuli predicting adverse outcomes.^72^ In contrast, BNST-AM cells develop *excitatory* responses to the same conditioned stimuli.^72^ Importantly, BNST-AL and AM neurons also display inverse activity changes in relation to the expression of contextual fear.^72^

The region-specific regulation of neuronal excitability we observed in BNST is consistent with these prior findings. The synaptic excitability of the non-CRF BNST-AL neurons was lower in PTSD-like rats, in keeping with the fact that these cells are inhibited during cued and contextual fear.^72^ Opposite to BNST-AL, BNST-AM and AV neurons had a higher synaptic excitability in PTSD-like rats, consistent with the higher firing rates of BNST-AM cells during cued and contextual fear.^72^ Interestingly, BNST-AL sends purely GABAergic projections to BNST-AM and AV,^73^ providing a potential substrate for the reciprocal activity fluctuations seen between these two BNST-A regions during fear and anxiety.

In the amygdala, we observed, robust phenotypic differences in the excitability of CeA neurons but they had an opposite polarity in CeL and CeM. In CeL, the amplitude and slope of BL-evoked EPSPs was higher in PTSD-like rats whereas the opposite was observed in CeM. Although CeL cells send GABAergic projections to CeM,^39, 57^ IPSP amplitudes did not differ between the two phenotypes in CeM. A possible explanation for this apparent contradiction is that CeL axons end distally in the dendrites of CeM neurons, preventing us from detecting changes in IPSP amplitudes with somatic recordings. Consistent with this possibility, distal GABAergic synapses to CeA neurons have a lower unitary conductance than somatic inhibitory synapses.^74^

While we observed differences in the efficacy of glutamatergic synapses onto BNST-AM, BNST-AV and CeL neurons, in these three cases PPF properties did not differ as a function of the rats’ phenotype. Thus, it is likely that postsynaptic factors, such as a change in the number and/or biophysical properties of AMPA receptors, are involved. Consistent with this, we found that CeL neurons had an increased sensitivity to uncaged glutamate in PTSD-like rats. More work will be needed to characterize these changes such as comparing AMPA/NMDA ratios and activity-dependent plasticity between phenotypes. Also to be identified are the signaling pathways that support the persistent changes in synaptic transmission we observed.

### Relevance to PTSD

Supporting the relevance of our data for understanding PTSD, there are many precedents in the literature for the participation of CeA and BNST in anxiety disorders.^75, 76, 77, 78, 79^ For instance, functional imaging studies in humans have reported increased BNST activation during the anticipation of adverse events and in stress-related anxiety disorders.^80, 81^ Moreover, in a non-human primate model of childhood dispositional anxiety, a temperament associated with an increased risk of developing mood disorders, CeA and BNST were found to be more active in monkeys with high- than low-dispositional anxiety^75, 76^ and CeA lesions decreased dispositional anxiety.^76^ Interestingly, the peptide PACAP (pituitary adenylate cyclase-activating polypeptide), which is enriched in CeL,^82^ enhances fear expression when infused in CeA^83^ and predicts PTSD symptom severity in women,^84^ causes a postsynaptic increase in the responses of CeL neurons to BL inputs,^85^ as we observed here in PTSD-like rats. PACAP inputs to CeL originate in the parabrachial nucleus, which also projects to BNST-AL.^86^ However, PACAP inputs to BNST-AL are concentrated in the oval nucleus where they target CRF neurons.^87^ Given the rapidly accumulating evidence implicating PACAP and CRF signaling in PTSD,^88^ it will be important to characterize the excitability of CRF neurons of the oval nucleus in the Lewis rat model of PTSD.

### Significance for the pathophysiology of PTSD

**Figure 5:**
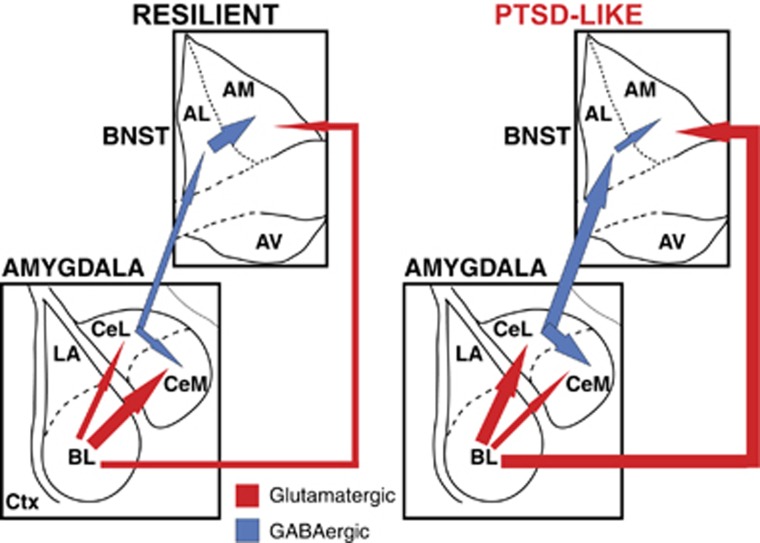
Differences in synaptic responsiveness between resilient (left) and PTSD-like (right) rats in the extended amygdala. Arrows of different thicknesses indicate relative strength or activity of the pathways depicted. The responsiveness of CeL neurons to BL inputs is higher in PTSD-like rats; that of CeM neurons is lower. In BNST, AL neurons are subjected to stronger inhibition in PTSD-like rats, whereas AM cells show a higher synaptic excitability. Given the GABAergic projections from CeL to CeM and BNST-AL, as well as from BNST-AL to BNST-AM, these differences should enhance the activity of BNST-AM neurons and reduce that of CeM cells. AL, anterolateral; AM, anteromedial; BL, basolateral; BNST, bed nucleus of the stria terminalis; CeL, central lateral; CeM, central medial; PTSD, post-traumatic stress disorder.

We observed distributed changes in synaptic responsiveness in a largely disconnected network. How would these changes impact the expression of fear and anxiety in an intact brain? CeL emerges as a key regulator in this context because it contributes GABAergic projections to CeM and BNST-AL but not BNST-AM.^31, 38^ Because CeL neurons are more excitable in PTSD-like rats, they should inhibit non-CRF BNST-AL neurons (Figure 5). Also, since BNST-AL contributes GABAergic projections to BNST-AM,^73^ BNST-AL inhibition by CeL inputs should cause a disinhibition of BNST-AM. In turn, higher activity levels in BNST-AM should increase, via its hypothalamic projections,^89^ anxiety. Last, because CeL sends GABAergic projections to CeM,^38, 39^ the PTSD-like state might be paradoxically associated with a reduced responsiveness of CeM fear output cells.

This model makes a startling prediction: in PTSD-like rats, control of fear expression is altered such that the influence of CeM is minimized whereas that of BNST-AM and AV is enhanced. This prediction could be tested by comparing the effects of BNST lesions on the expression of conditioned fear responses to cues in the two phenotypes. Indeed, prior studies found that cued fear is unaffected by BNST lesions.^61, 62, 64, 65^ However, our results predict that, after predatory threat, such interventions will reduce cued fear in PTSD-like but not resilient rats. If supported, this prediction might explain the greater resistance of conditioned fear to extinction training in PTSD-like rats:^11^ because mechanisms of fear expression would differ between the two phenotypes, so would extinction mechanisms. Also, given earlier findings indicating that BNST activity promotes fear generalization,^65^ the enhanced responsiveness of BNST-AM and AV neurons might promote generalization of fear to innocuous cues and contexts, a defining feature of anxiety disorders.
