# Supplementary material for: Altered responsiveness of BNST and amygdala neurons in trauma-induced anxiety
Source: Transl Psychiatry. 2016 Jul 19;6(7):e857–. doi: 10.1038/tp.2016.128 (PMC5545714; doi:10.1038/tp.2016.128)
Supplement: Supplementary Tables [file tp2016128x1.doc]

| **Table 1** | ***Incidence of BNST-A cell types in resilient and PTSD-like rats*** | | | | | |
| --- | --- | --- | --- | --- | --- | --- |
|  | BNST-AL | | BNST-AM | | BNST-AV | |
|  | Resilient | PTSD-like | Resilient | PTSD-like | Resilient | PTSD-like |
| Cell Type | % | % | % | % | % | % |
|  |  |  |  |  |  |  |
| LTB | 42.4 | 35.7 | 81.5 | 68 | 50 | 65.4 |
|  |  |  |  |  |  |  |
| RS | 42.4 | 46.4 | 14.8 | 20 | 29.2 | 23 |
|  |  |  |  |  |  |  |
| fIR | 12.1 | 10.7 | 3.7 | 12 | 0 | 3.8 |
|  |  |  |  |  |  |  |
| LF | 3 | 7.1 | 0 | 0 | 0 | 0 |
|  |  |  |  |  |  |  |
| SA | 0 | 0 | 0 | 0 | 20.8 | 7.7 |
|  |  |  |  |  |  |  |

| **Table 2** | ***Physiological Properties of RS cells in BNST-AL* (values are means ± SEM)** | | | | | | | | |
| --- | --- | --- | --- | --- | --- | --- | --- | --- | --- |
|  |  | | | | | Action Potential | | |  |
|  | n | Rest (mV) | Rin (MΩ) | Time Constant (ms) | Threshold (mV) | | Amplitude (mV) | Duration (ms) | |
|  |  |  |  |  |  | |  |  | |
| Resilient | 13 | -64 ± 1.2 | 563.9 ± 39.4 | 23.2 ± 2.4 | -41.4 ± 1.7 | | 84.5 ± 2 | 1.05 ± 0.05 | |
|  |  |  |  |  |  | |  |  | |
| PTSD-like | 14 | -64.7 ± 1.1 | 562.7 ± 30 | 22.3 ± 3.1 | -42.5 ± 0.9 | | 79.7 ± 1.2 | 1.17 ± 0.03 | |
|  |  |  |  |  |  | |  |  | |

| **Table 3** | ***Physiological Properties of LTB cells in BNST-AL* (values are means ± SEM)** | | | | | | | | |
| --- | --- | --- | --- | --- | --- | --- | --- | --- | --- |
|  |  | | | | | Action Potential | | |  |
|  | n | Rest (mV) | Rin (MΩ) | Time Constant (ms) | Threshold (mV) | | Amplitude (mV) | Duration (ms) | |
|  |  |  |  |  |  | |  |  | |
| Resilient | 10 | -63.67 ± 1.2 | 577.6 ± 55.9 | 22.7 ± 2.9 | -42 ± 1.4 | | 79.7 ± 1.5 | 1.29 ± 0.1 | |
|  |  |  |  |  |  | |  |  | |
| PTSD-like | 14 | -62.17 ± 1.5 | 613.4 ± 50.8 | 20.6 ± 1.8 | -41.4 ± 1.2 | | 78.2 ± 1.3 | 1.09 ± 0.06 | |
|  |  |  |  |  |  | |  |  | |

| **Table 4** | ***Physiological Properties of RS cells in BNST-AM* (values are means ± SEM)** | | | | | | | | |
| --- | --- | --- | --- | --- | --- | --- | --- | --- | --- |
|  |  | | | | | Action Potential | | |  |
|  | n | Rest (mV) | Rin (MΩ) | Time Constant (ms) | Threshold (mV) | | Amplitude (mV) | Duration (ms) | |
|  |  |  |  |  |  | |  |  | |
| Resilient | 4 | -59 ± 4 | 855 ± 144.6 | 33.9 ± 4.8 | -38.5 ± 3.6 | | 69.9 ± 1.1 | 1.07 ± 0.31 | |
|  |  |  |  |  |  | |  |  | |
| PTSD-like | 5 | -53.1 ± 4.5 | 725.2 ± 229 | 40.9 ± 8.4 | -42.4 ± 2 | | 73.7 ± 2.3 | 1.38 ± 0.17 | |
|  |  |  |  |  |  | |  |  | |

| **Table 5** | ***Physiological Properties of LTB cells in BNST-AM* (values are means ± SEM)** | | | | | | | | |
| --- | --- | --- | --- | --- | --- | --- | --- | --- | --- |
|  |  | | | | | Action Potential | | |  |
|  | n | Rest (mV) | Rin (MΩ) | Time Constant (ms) | Threshold (mV) | | Amplitude (mV) | Duration (ms) | |
|  |  |  |  |  |  | |  |  | |
| Resilient | 22 | -55 ± 1.3 | 674.1 ± 54.4 | 34.5 ± 3.6 | -39.5 ± 0.8 | | 76.2 ± 0.8 | 1.45 ± 0.12 | |
|  |  |  |  |  |  | |  |  | |
| PTSD-like | 17 | -58.9 ± 1.5 | 692.8 ± 79.2 | 36.4 ± 3.5 | -39.2 ± 1.1 | | 75.8 ± 1 | 1.39 ± 0.08 | |
|  |  |  |  |  |  | |  |  | |

| **Table 6** | ***Physiological Properties of RS cells in BNST-AV* (values are means ± SEM)** | | | | | | | | |
| --- | --- | --- | --- | --- | --- | --- | --- | --- | --- |
|  |  | | | | | Action Potential | | |  |
|  | n | Rest (mV) | Rin (MΩ) | Time Constant (ms) | Threshold (mV) | | Amplitude (mV) | Duration (ms) | |
|  |  |  |  |  |  | |  |  | |
| Resilient | 7 | -56.2 ± 5.4 | 780.6 ± 114.6 | 42.8 ± 5.5 | -39 ± 2.8 | | 73.4 ± 1.8 | 1.22 ± 0.16 | |
|  |  |  |  |  |  | |  |  | |
| PTSD-like | 6 | -51.5 ± 1.1 | 887.8 ± 128.2 | 31.6 ± 3.2 | -36 ±1.9 | | 75.1 ± 2.2 | 1.11 ± 0.16 | |
|  |  |  |  |  |  | |  |  | |

| **Table 7** | ***Physiological Properties of LTB cells in BNST-AV* (values are means ± SEM)** | | | | | | | | |
| --- | --- | --- | --- | --- | --- | --- | --- | --- | --- |
|  |  | | | | | Action Potential | | |  |
|  | n | Rest (mV) | Rin (MΩ) | Time Constant (ms) | Threshold (mV) | | Amplitude (mV) | Duration (ms) | |
|  |  |  |  |  |  | |  |  | |
| Resilient | 12 | -57.8 ± 1.67 | 621.8 ± 69.3 | 31.2 ± 1.8 | -37.9 ± 1.8 | | 74.0 ± 1 | 1.1 ± 0.07 | |
|  |  |  |  |  |  | |  |  | |
| PTSD | 17 | -56.3 ± 1.81 | 807.5 ± 59 | 35.2 ± 4.2 | -39 ± 1 | | 74.8 ± 1 | 1.29 ± 0.09 | |
|  |  |  |  |  |  | |  |  | |

| **Table 8.** | ***Physiological Properties of BL neurons*** | | | | | |
| --- | --- | --- | --- | --- | --- | --- |
|  |  |  | Action Potential | | |  |
|  | Rest, mV | R_in_, MΩ | Threshold, mV | Amplitude, mV | Duration, ms | Time Constant, ms |
| Resilient | -67.9 ± 0.6 | 128.3 ± 8.1 | -40.9 ± 0.6 | 90.7 ± 1.5 | 0.85 ± .02 | 40.8 ± 2.2 |
|  |  |  |  |  |  |  |
| PTSD-like | -67.5 ± 0.6 | 135.3 ± 6.9 | -40.9 ± 0.5 | 91.8 ± 1.7 | 0.87 ± .02 | 38.4 ± 2.9 |
|  |  |  |  |  |  |  |

Values are means ± SE. BL, basolateral nucleus of the amygdala; Rest, resting potential; R_in_, input resistance.

| **Table 9.** | | ***Physiological Properties of CeL neurons*** | | | | | | | | | |
| --- | --- | --- | --- | --- | --- | --- | --- | --- | --- | --- | --- |
|  |  | |  | | Action Potential | | | |  | |  |
|  | | Rest, mV | | R_in_, MΩ | | Threshold, mV | Amplitude, mV | Duration, ms | | Time Constant, ms | |
| Resilient | | -62.8 ± 0.7 | | 414.7 ± 22.8 | | -39.5 ± 0.8 | 84.2 ± 1.3 | 1.00 ± .03 | | 42.8 ± 2.8 | |
|  | |  | |  | |  |  |  | |  | |
| PTSD-like | | -63.9 ± 0.8 | | 373.4 ± 17.7 | | -38.3 ± 1.4 | 84.1 ± 1.2 | 1.05 ± .03 | | 43.2 ± 2.5 | |
|  | |  | |  | |  |  |  | |  | |

Values are means ± SE. CeL, central lateral amygdala; Rest, resting potential; R_in_, input resistance.

| **Table 10.** | | ***Physiological Properties of CeM neurons*** | | | | | | | | | |
| --- | --- | --- | --- | --- | --- | --- | --- | --- | --- | --- | --- |
|  |  | |  | | Action Potential | | | |  | |  |
|  | | Rest, mV | | R_in_, MΩ | | Threshold, mV | Amplitude, mV | Duration, ms | | Time Constant, ms | |
| Resilient | | -63.8 ± 1.3 | | 450.9 ± 62.1 | | -42.1 ± 1.5 | 79.5 ± 2.1 | 0.84 ± .05 | | 31.8 ± 2.6 | |
|  | |  | |  | |  |  |  | |  | |
| PTSD-like | | -62.1 ± 1.0 | | 472.9 ± 29.8 | | -44.7 ± 0.9 | 84.5 ± 1.8 | 0.75 ± .03 | | 31.6 ± 2.3 | |
|  | |  | |  | |  |  |  | |  | |
| **Table 11.** | | ***Physiological Properties of LTB neurons*** | | | | | | | | | |
|  |  | |  | | Action Potential | | | |  | |  |
|  | | Rest, mV | | R_in_, MΩ | | Threshold, mV | Amplitude, mV | Duration, ms | | Time Constant, ms | |
| Resilient | | -61.9 ± 1.5 | | 454.5 ± 51.2 | | -46.0 ± 0.9 | 79.2 ± 2.6 | 0.91 ± .08 | | 31.3 ± 3.6 | |
|  | |  | |  | |  |  |  | |  | |
| PTSD-like | | -64.5 ± 1.7 | | 490.0 ± 42.0 | | -45.5 ± 1.1 | 80.5 ± 3.1 | 0.84 ± .06 | | 29.4 ± 2.2 | |
|  | |  | |  | |  |  |  | |  | |
| **Table 12.** | | ***Physiological Properties of RS neurons*** | | | | | | | | | |
|  |  | |  | | Action Potential | | | |  | |  |
|  | | Rest, mV | | R_in_, MΩ | | Threshold, mV | Amplitude, mV | Duration, ms | | Time Constant, ms | |
| Resilient | | -66.8 ± 2.4 | | 575.1 ± 108 | | -37.4 ± 3.0 | 80.7 ± 3.9 | 0.76 ± .05 | | 32.6 ± 4.4 | |
|  | |  | |  | |  |  |  | |  | |
| PTSD-like | | -60.6 ± 1.3 | | 504.6 ± 39.6 | | -43.7 ± 1.1 | 84.7 ± 2.2 | 0.73 ± .02 | | 35.2 ± 3.4 | |
|  | |  | |  | |  |  |  | |  | |
