# Supplementary material for: Altered responsiveness of BNST and amygdala neurons in trauma-induced anxiety
Source: Transl Psychiatry. 2016 Jul 19;6(7):e857–. doi: 10.1038/tp.2016.128 (PMC5545714; doi:10.1038/tp.2016.128)
Supplement: Supplementary Information [file tp2016128x2.doc]

**SUPPLEMENTARY METHODS**

**Elevated plus maze**

The EPM is a commonly used behavioral assay of anxiety in rodents. Our EPM is elevated 60 cm from the floor and consists of four arms, 50 cm long by 10 cm wide. Two of the arms are enclosed by black walls, 30 cm in height, and two of the arms are open, with a white Plexiglas floor.

**Preparation of brain slices and whole-cell recordings**

Solution used for perfusion and sectioning of the brains: This solution contained (in mM): 248 sucrose, 2.5 KCl, 7 MgCl_2_, 23 NaHCO_3_, 1.2 NaH_2_PO_4_, 7 glucose.

Control aCSF: This solution contained (in mM) 124 NaCl, 2.5 KCl, 1.25 NaH_2_PO_4_, 26 NaHCO_3_, 1 MgCl_2_, 2 CaCl_2_, 10 glucose. (pH 7.3, 300 mOsm).

Intracellular solution: This solution contained (in mM): 130 K-gluconate, 10 N-2-hydroxyethylpiperazine-N'-2-ethanesulfonic acid, 10 KCl, 2 MgCl_2_, 2 ATP-Mg, and 0.2 GTP-tris(hydroxy-methyl)aminomethane (pH 7.2, 280 mOsm).

Whole-cell recordings were obtained under visual guidance using differential interference contrast and infrared video-microscopy and pipettes (7-10 MΩ) pulled from borosilicate glass capillaries. Recordings were obtained with an Axoclamp-2B amplifier and digitized at 10 kHz with a Digidata-1200 interface (Axon Instruments, Foster City, CA).

**Criteria used to distinguish, direct, orthodromic, and antidromic action potentials**

These three kinds of spikes can be easily distinguished using the following standard criteria. Direct spikes arise directly from the stimulation artifact. In contrast, anti- and orthodromic spikes are triggered after a delay. Antidromic spikes can be distinguished from orthodromic action potentials because the former have a fixed latency whereas the latter ones show jitter.
