# Supplementary figures and images for: Altered responsiveness of BNST and amygdala neurons in trauma-induced anxiety
Source: Transl Psychiatry. 2016 Jul 19;6(7):e857–. doi: 10.1038/tp.2016.128 (PMC5545714; doi:10.1038/tp.2016.128)

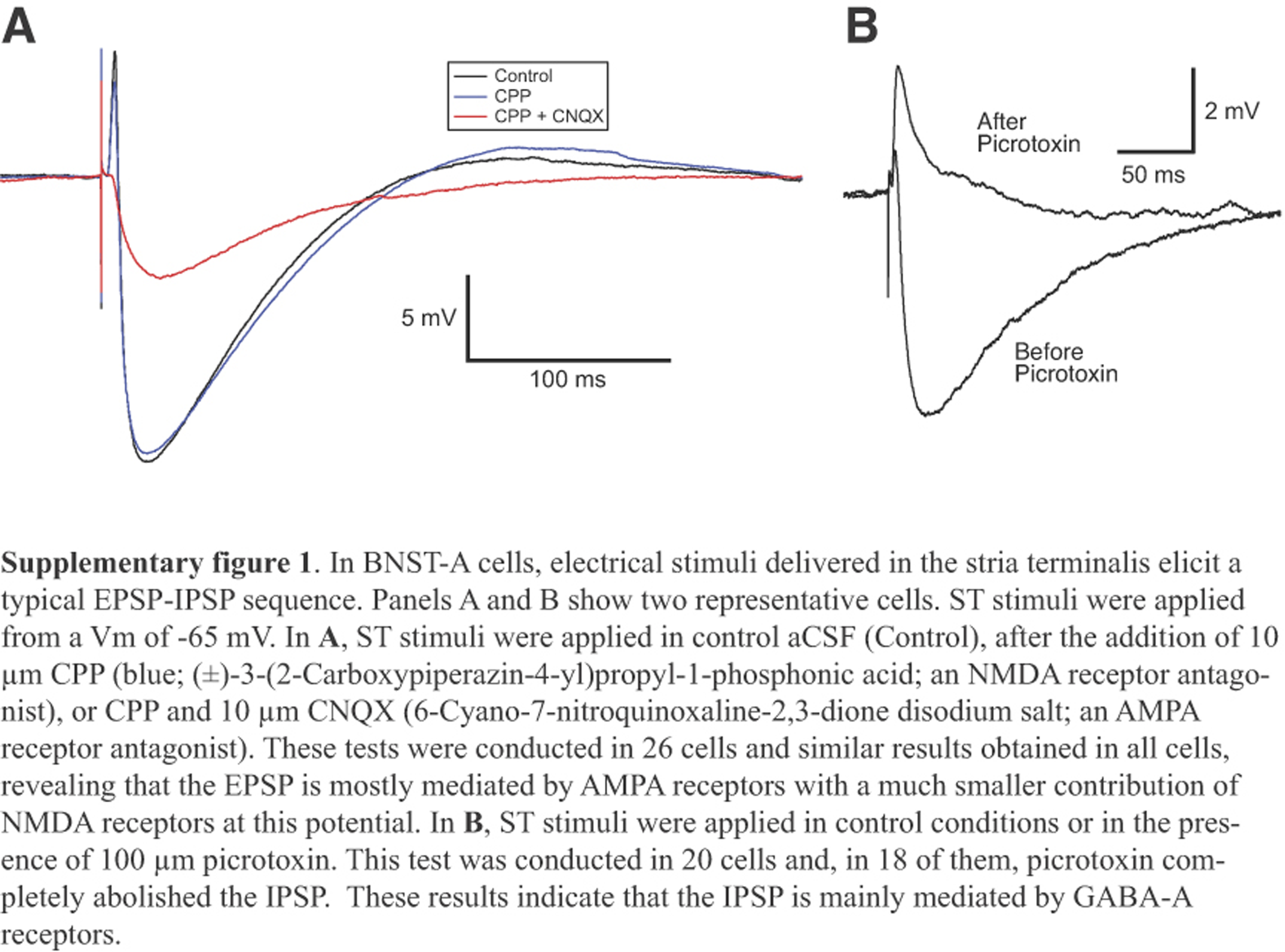

Supplement: Supplementary Figure 1 [file tp2016128x3.tif]

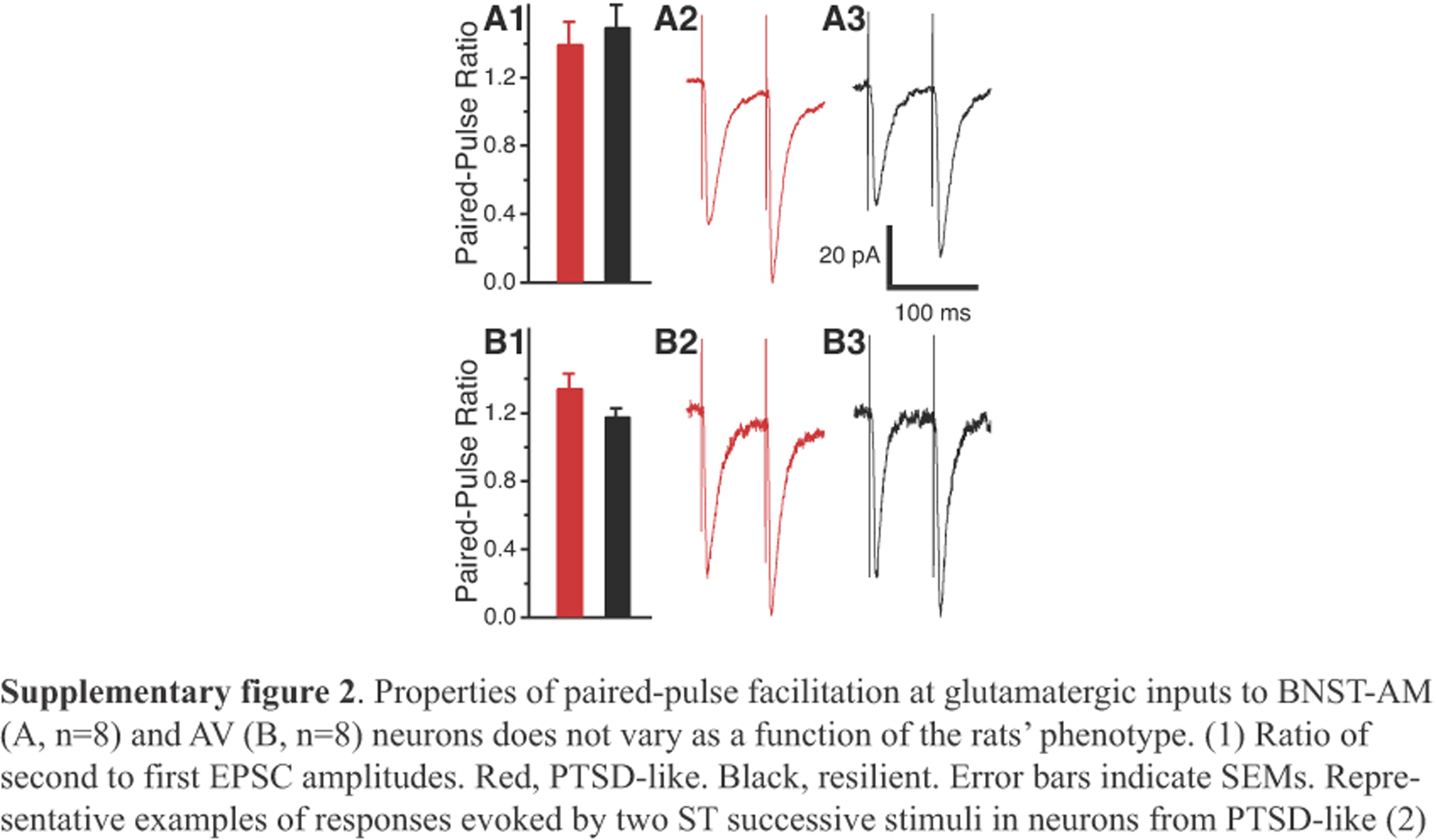

Supplement: Supplementary Figure 2 [file tp2016128x4.tif]

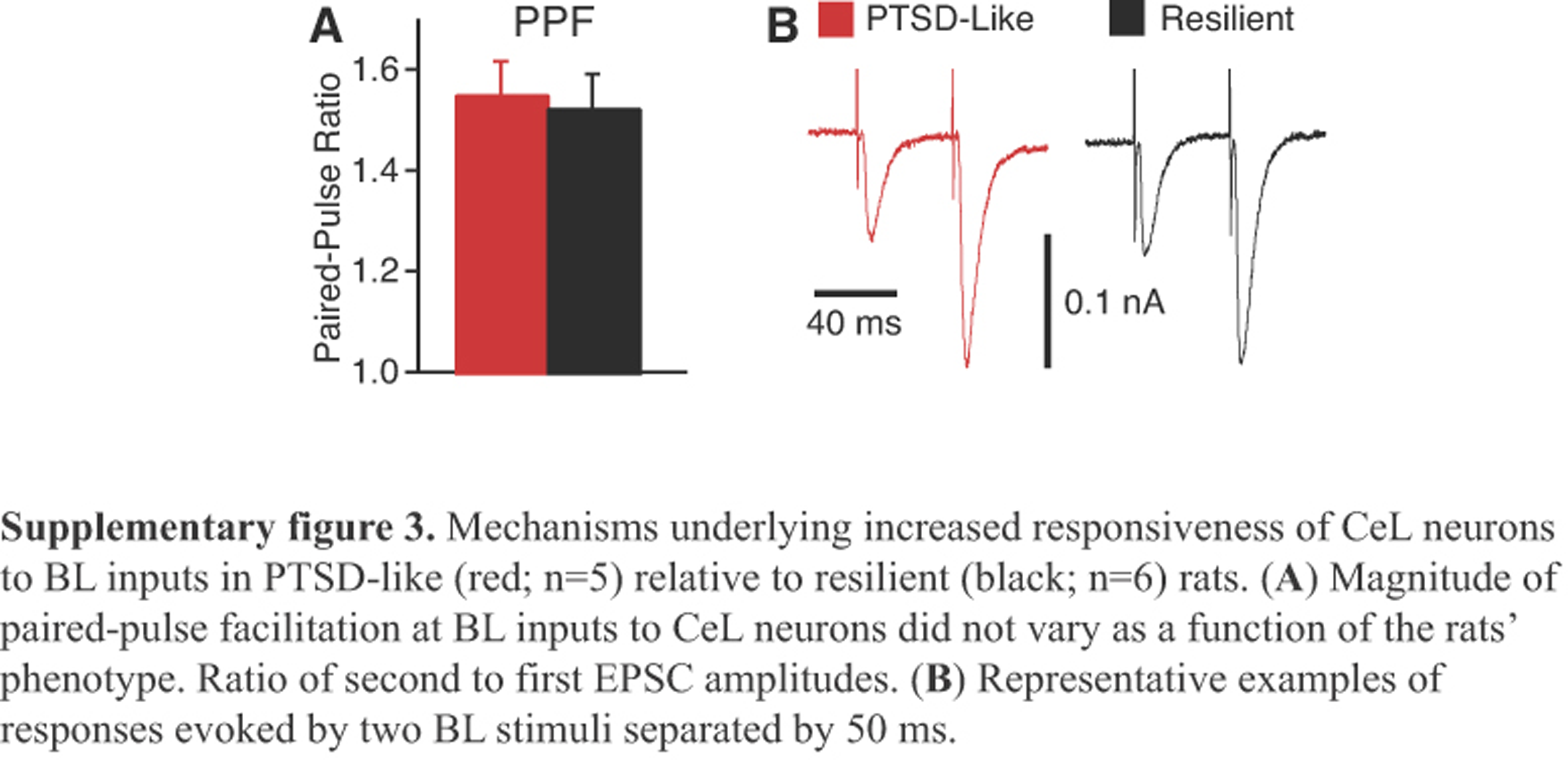

Supplement: Supplementary Figure 3 [file tp2016128x5.tif]
